# Predictors of Non-invasive Ventilation Failure and Associated Factors Among the COVID-19 Patients Admitted to Intensive Care Unit

**DOI:** 10.5812/aapm-140847

**Published:** 2023-12-12

**Authors:** Hesam Aldin Varpaei, Nurhan Bayraktar, Mostafa Mohammadi

**Affiliations:** 1 College of Nursing, Michigan State University, East Lansing, USA; 2 Atilim University School of Health Sciences, Nursing Department, Ankara, Turkiye; 3 Department of Critical Care, Imam Khomeini Hospital Complex, Tehran University of Medical Sciences, Tehran, Iran

**Keywords:** Critical Care, Noninvasive Ventilation, Acute Respiratory Distress Syndrome, Airway Management, Nursing Care

## Abstract

**Background:**

Non-invasive ventilation (NIV) is a method of oxygenation supply that eliminates the need for an endotracheal airway. Non-invasive ventilation failure is defined as the necessity for endotracheal intubation or death during the NIV trial.

**Objectives:**

This study aimed to identify the predictors and associated factors of NIV failure in coronavirus disease 2019 (COVID-19) patients admitted to an intensive care unit (ICU).

**Methods:**

This retrospective, longitudinal cohort study utilized electronic medical records of COVID-19 patients admitted to the ICU. A total of 150 patients were included in the study. Patient demographics, medical history, laboratory tests, partial pressure of carbon dioxide (PCO_2_), oxygen saturation (SpO_2_), heart rate, acidosis, consciousness, oxygenation, and respiratory rate (HACOR score), and the ratio of oxygen saturation (ROX) index (the SpO_2_/fraction of inspired oxygen [FIO_2_] to respiratory rate [SF] ratio) were recorded. Non-invasive ventilation failure was determined based on the need for endotracheal intubation or cardiac-respiratory arrest while on NIV.

**Results:**

Of 150 patients, 55.3% were male (mean age: 55.9 years), with an NIV failure rate of 67.3%, a mortality rate of 66.7%, and 3.3% of patients requiring tracheostomy after NIV failure. The ROX index consistently decreased over time, and an increase in the HACOR score and PCO2 after 6 hours of commencing NIV were the predictors of NIV failure. Additionally, higher levels of lactate dehydrogenase, lower SF ratios, and higher APACHE scores upon ICU admission were significantly associated with NIV failure. Notably, the erythrocyte sedimentation rate (ESR) as an inflammatory index, SF ratio upon ICU admission, HACOR score, ROX index, and PCO2 after 12 hours were significant predictors of in-hospital mortality in patients receiving NIV.

**Conclusions:**

The ROX index, HACOR scale, and PCO_2_ are significant predictors of both NIV failure and in-hospital mortality.

## 1. Background

Non-invasive ventilation (NIV) is a method of oxygenation supply that utilizes various interfaces such as a total face mask, nasal mask, helmet, nasal pillows, and oral mask, eliminating the need for an endotracheal airway (1). It is considered an effective treatment for various pulmonary diseases (2, 3) and acute hypercapnic respiratory failure, such as chronic obstructive pulmonary disease (COPD) exacerbation (4).

Non-invasive ventilation failure is defined as the need for endotracheal intubation (ETI) or death (5). We use the term “NIV failure” when a patient who has received NIV subsequently requires intubation. Its prevalence ranges from 5% to 60%, depending on various factors, including the underlying cause of acute respiratory failure (ARF) (6). For example, NIV failure rates were reported as 20% in cases of community-acquired pneumonia (CAP) (7), 26% (8), and 51.9% (9) in other contexts.

Coronavirus disease 2019 (COVID-19), the focus of this paper, has resulted in thousands of deaths worldwide due to respiratory failure. The use of NIV to treat the hypoxemic state of COVID-19 patients has been widely discussed (10), and it is hypothesized that NIV failure is more common in COVID-19 patients than in those with COPD, CAP, or ARF. Non-invasive ventilation failure is associated with increased mortality in patients experiencing respiratory distress (11) and is linked to longer hospital stays, which can be costly for both patients and healthcare systems. Another potential negative consequence of NIV trials is scarring (ulcers) in the NIV interfaces (12). The improper use of NIV in non-designated areas has been associated with a high mortality rate (12).

Oxygen therapy and delivering appropriate care are two crucial responsibilities of intensive care unit (ICU) nurses. In ICUs, nurses closely monitor patients and are responsible for reporting any significant changes that could endanger a patient’s life (13). According to a study (14), a nurse-driven NIV protocol can reduce NIV failure by 15%, prevent intubation by 15%, and reduce mortality by 5%. The ICU nurses play a significant role in patient monitoring and in minimizing environmental barriers to optimize ventilation and oxygenation during NIV (15, 16). Therefore, their practice and knowledge of NIV (17) are of great importance.

Nursing care in ICUs can be particularly challenging, especially during times of crisis, such as the COVID-19 pandemic. Due to the high workload, nurses might not have sufficient time to closely monitor patients for NIV failure. The identification of predictive factors of NIV failure allows nurses to take appropriate action promptly. It also enables them to classify patients as “high risk for NIV failure” and assign the most experienced nurses to provide care. The experiences of the COVID-19 pandemic underscore the need for a more precise understanding of its pathology and consequences. Currently, there is no well-designed original report from Iran regarding NIV failure and associated factors.

## 2. Objectives

The present study aimed to determine the rate of NIV failure and associated factors among COVID-19 patients admitted to the ICU.

## 3. Methods

### 3.1. Study Design

This study was a retrospective, longitudinal cohort study. 

### 3.2. Study Setting

This study was conducted using the electronic medical data from Imam Khomeini Hospital Complex (IKHC), Tehran, Iran. 

At the time of this study, there was no integrated institutional protocol for the use of NIV in the ICU for COVID-19 patients. The utilization of NIV was based on the clinical judgment of physicians. Standard monitoring for COVID-19 patients under NIV included continuous monitoring of heart rate, respiratory rate, pulse oximeter, non-invasive blood pressure, and arterial blood gas (ABG) levels every 2 hours.

### 3.3. Sample

The study sample consisted of COVID-19 patients admitted to the ICU who required NIV within March 2021 to July 2022. All patients meeting the inclusion criteria were included in this study. The final sample size for the study was 150 patients. The accessible population for this study comprised critically ill COVID-19 patients, both male and female (adults), in ICUs who required NIV. All patients should have had a confirmed real-time polymerase chain reaction (PCR) test for severe acute respiratory syndrome coronavirus 2 (SARS-CoV-2) or a rapid antigen test for SARS-CoV-2, or they should have exhibited significant signs and symptoms of the disease, approved by a physician for COVID-19.

The inclusion criteria were patients aged between 18 and 80 years with no history of blood dyscrasias or lung fibrosis. The exclusion criteria included pregnant patients, recently extubated patients, and patients in the end-stage of cancer.

### 3.4. Study Tools

The data were collected through a researcher-designed questionnaire that included patients’ demographics, past medical history, laboratory tests at the time of ICU admission, oxygen saturation (SpO_2_), ABGs, including partial pressure of oxygen (PaO_2_), and partial pressure of carbon dioxide (PCO_2_) at admission, 6 hours, and 12 hours after NIV initiation, vital signs, nursing observations regarding the presence of facial ulcers, Richmond Agitation-Sedation Scale (RASS) (18), APACHE II (Acute Physiology And Chronic Health Evaluation II) score (19), HACOR (heart rate, acidosis, consciousness, oxygenation, and respiratory rate) score (20), the ratio of oxygen saturation (ROX) index (21), Glasgow Coma Scale (GCS) (22), and final outcome (discharged or expired). All these tools are standardized scales that have previously been proven to be reliable and are available in the standard English version without the need for translation or reliability checking.

The GCS is a 15-point scale used to assess consciousness, developed by Dr. Bryan Jennett and Dr. Graham Teasdale in 2000. Glasgow Coma Scale scores less than 10, 7, and 4 indicate loss of consciousness, a coma, and a deep coma, respectively. Patients with scores ranging from 3 to 8 are considered to be in a coma.

The APACHE II score, developed by Dr. William Knaus in 1970, estimates ICU mortality based on a set of laboratory data and patient symptoms, taking into consideration both acute and chronic illnesses. The data used should be from the first 24 hours in the ICU, with the worst value (farthest from baseline/normal) being used. APACHE-II is categorized as a Likert scale, with scores from 0 - 4, 5 - 9, 10 - 14, 15 - 19, and 20 - 24 predicting 4%, 8%, 15%, 25%, and 40% mortality, respectively.

The RASS, developed by Dr. Curtis Sessler in 2000, assesses the level of sedation and agitation. Richmond Agitation-Sedation Scale Likert scores range from - 5 (unarousable) to + 4 (combative).

The ROX index, developed in 1999, is calculated as follows:

ROX index = (SpO_2 _/FiO_2_)/(respiratory rate [RR])

The HACOR scale was developed by Duan J et al. in 2017. The HACOR scale induces 5 parameters, including heart rate, respiratory rate, GCS, PF ratio, and arterial PH. According to this tool, each component will be allocated a score for the final calculation.

### 3.5. Data Collection

**Figure 1.**
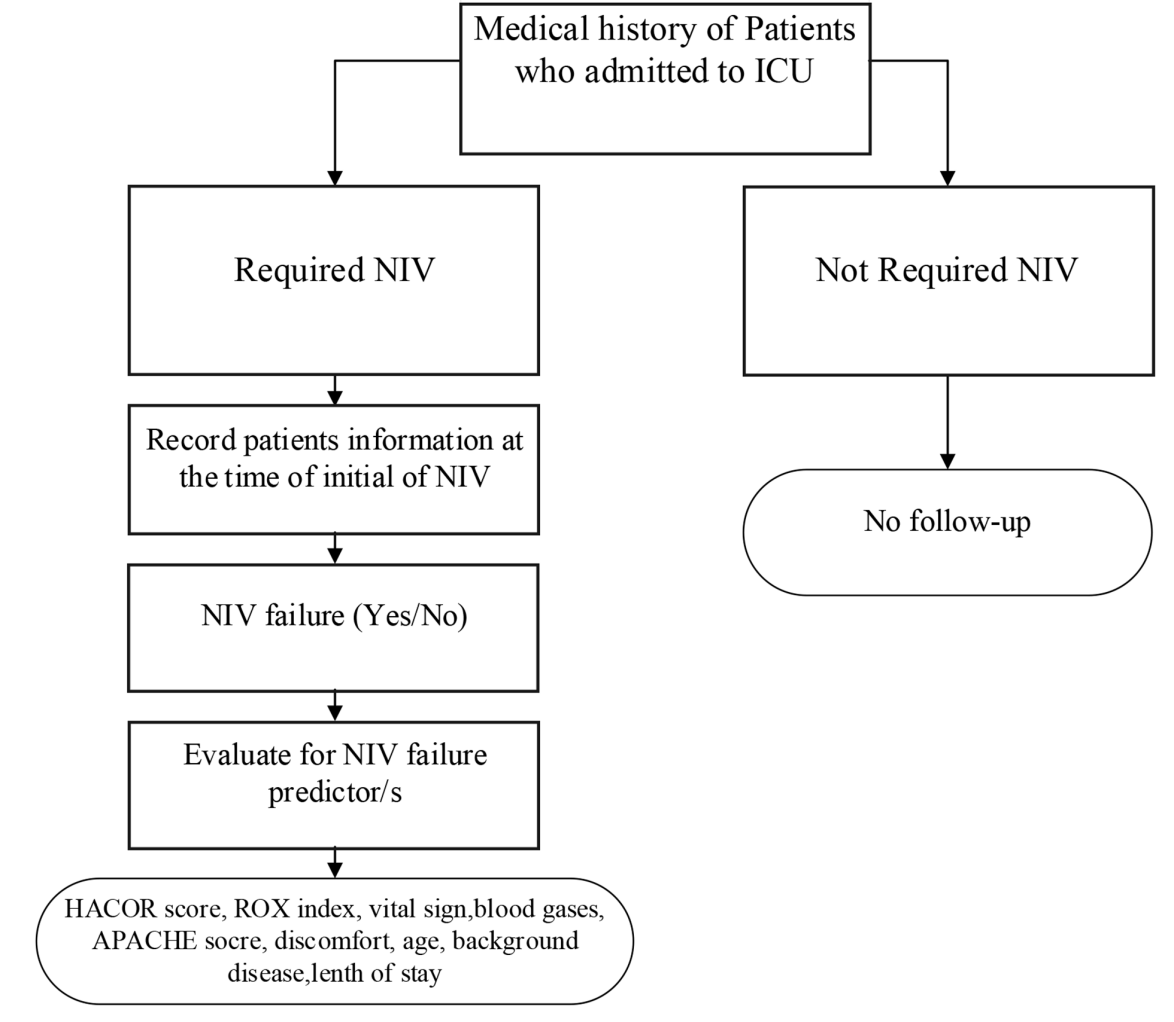
Flowchart of the Study; (Abbreviations: ICU, intensive care unit; NIV, non-invasive ventilation; ROX, ratio of oxygen saturation)

The data were collected retrospectively from the electronic records of ICU patients within March 2021 and July 2022 by the researchers (Figure 1). Patients who met the inclusion criteria were included in this study, and the necessary information was collected by the researchers. Vital signs, laboratory tests, and blood gases were extracted from medical records. Patients’ demographic data and vital signs were recorded at the time of NIV initiation. Oxygenation indexes, including the ROX index, HACOR score, PCO_2_, and SpO_2_, were collected at three-time points (i.e., at the start of NIV, 6 hours after NIV initiation, and 12 hours after NIV initiation).

Patients’ vital signs, laboratory tests, and blood gases were collected from the medical records. The occurrence of NIV failure (according to the definition), length of ICU stays, outcomes, and facial ulcers (according to nursing reports of facial ulcers) were also collected from patients’ medical records. The patients’ electronic data were assessed for eligibility to be included in this study. Patients’ laboratory and oxygenation data (ROX, HACOR, PCO_2_, SpO_2_) at the time of NIV initiation in the ICU were recorded. Oxygenation data (ROX, HACOR, PCO_2_, and SpO_2_) were recorded at 6 and 12 hours after NIV initiation. The patients were followed up on for outcomes until the end of their ICU stay.

The criteria for NIV failure were the need for endotracheal tube insertion (intubation) or cardiac-respiratory arrest while on NIV.

### 3.6. Ethical Considerations

Ethical approval was obtained from the Institutional Review Board (IRB: YDU/2022/104-1577) of Near East University in Turkey before conducting the study. Additionally, confirmation was obtained from the IKHC and the head of the ICU.

### 3.7. Outcomes

The primary outcome of this study was NIV failure, and the secondary outcome was in-hospital mortality or discharge.

### 3.8. Statistical Analysis

Statistical analysis was performed using SPSS software (version 26). P value less than 5% was considered to reject the null hypothesis. All continuous data were first analyzed for parametric assumptions using the Kolmogorov-Smirnov test. The data that met parametric assumptions were analyzed using an independent t-test; nevertheless, the Mann-Whitney U test was used if the parametric assumptions were not met. The binary logistic test was employed to predict NIV failure and mortality.

## 4. Results

A total of 150 patients were included in this study, with 55.3% being male (mean age: 55.9 ± 13.48 years, body mass index [BMI]: 26.13 ± 5.16 kg/m^2^). Regarding comorbidities, hypertension (34.7%), diabetes (28.7%), and ischemic heart disease (16.7%) were the most common comorbidities, respectively (Table 1). 

**Table 1.** Comparison of Predictors and Associated Factors with Non-invasive Ventilation (NIV) Failure Status ^a^

Clinical Variables	Overall (N = 150)	NIV Failure		Test Statistics	P-Value
		**No (N = 49)**	**Yes (N = 101)**		
**Age (y)**	55.90 (13.48)	52.10 ± 13.96	57.74 ± 12.91	U = 1865.5	0.015
**APACHE II**	21.34 (3.12)	18.61 ± 1.96	23.5 ± 2.60	U = 755.5	< 0.001
**BMI (kg/m** ^**2**^ **)**	26.13 (5.16)	25.83 ± 3.97	26.28 ± 5.66	U = 2389.5	0.733
**ROX baseline**	4.01 (1.22)	4.71 ± 1.19	3.67 ± 1.09	U = 1236.5	< 0.001
**ROX after 6 hours**	4.00 (1.15)	5.11 ± 1.10	3.46 ± 0.70	U = 479.5	< 0.001
**ROX after 12 hours**	3.97 (1.20)	5.28 ± 1.04	3.33 ± 0.62	U = 158.5	< 0.001
**HACOR baseline**	8.68 (2.28)	7.33 ± 1.53	9.34 ± 2.30	U = 1117.0	< 0.001
**HACOR after 6 hours**	7.81 (1.63)	6.53 ± 1.00	8.43 ± 1.51	U = 751.5	< 0.001
**HACOR after 12 hours**	8.81 (2.47)	6.37 ± 1.03	10.00 ± 2.06	U = 242.5	< 0.001
**Pulse rate (per min)**	94.52 (20.61)	87.57 ± 15.31	97.89 ± 22.04	U = 1843.5	0.011
**Respiratory rate (per min)**	28.91 (7.46)	24.94 ± 6.67	30.84 ± 7.08	U = 1326.0	< 0.001
**Systolic blood pressure (mm Hg)**	122.38 (17.63)	120.14 ± 14.02	123.47 ± 19.11	U = 2159.0	0.206
**GSC**	14.35 (1.01)	14.76 ± 0.52	14.15 ± 1.13	U = 1696.0	< 0.001
**RASS**	- 0.96 (1.36)	- 1.33 ± 1.26	- 0.78 ± 1.38	U = 1872.5	0.008
**Reported pain intensity (VAS)**	1.81 (1.86)	1.06 ± 1.83	2.18 ± 1.77	U = 1552.5	< 0.001
**SpO_2_**	86.62 (6.44)	88.69 ± 5.09	85.61 ± 6.80	U = 1787.0	0.006
**SpO_2_-2**	88.17 (6.02)	91.90 ± 3.37	86.37 ± 6.20	U = 992.0	< 0.001
**SpO_2_-3**	89.00 (6.09)	93.59 ± 3.18	86.77 ± 5.92	U = 642.5	< 0.001
**PCO_2_-1**	43.21 (10.10)	39.68 ± 9.08	44.92 ± 10.17	U = 1712.0	0.002
**PCO_2_-2**	43.11 (8.20)	39.78 ± 7.65	44.73 ± 8.01	U = 1599.5	< 0.001
**PCO2-3**	42.44 (9.03)	35.58 ± 5.77	45.77 ± 8.43	U = 657.0	< 0.001
**CRP (mg/dl)**	91.31 (60.56)	77.67 ± 52.83	97.52 ± 63.03	U = 1901.0	0.078
**LDH (IU/lit)**	959.36 (345.41)	818.13 ± 306.18	1023.56 ± 344.67	U = 1054.5	< 0.001
**ESR (mm/h)**	58.12 (28.60)	63.90 ± 32.72	55.73 ± 26.54	U = 1711.0	0.278
**SF ratio**	108.21 (8.92)	110.78 ± 6.23	106.96 ± 9.75	U = 1859.0	0.013
**ICU stay (days)**	10.00 (7.21)	9.76 ± 5.38	10.12 ± 7.97	U = 2279.0	0.489
**Gender and comorbidities**					
Male	83 (55.3)	31 (63.3)	52 (51.5)	χ^2^ = 1.85	0.170
Female	67 (44.7)	18 (36.70)	49 (48.50)		
Diabetes mellitus	43 (28.7)	13 (26.50)	30 (29.70)	χ^2^ = 0.162	0.680
Cardiovascular diseases	18 (12.0)	3 (6.10)	15 (14.90)	χ^2^ = 2.381	0.120
Hypertension	52 (34.7)	14 (28.60)	38 (37.60)	χ^2^ = 1.194	0.270
Hypothyroidism	12 (8.0)	5 (10.20)	7 (6.90)	χ^2^ = 0.480	0.520
Chronic kidney disease	10 (6.7)	3 (6.10)	7 (6.90)	χ^2^ = 0.035	0.999
Asthma	6 (4.0)	2 (4.10)	4 (4.00)	χ^2^ = 0.001	0.999
COPD	1 (0.7)	0 (0.00)	1 (1.00)	χ^2^ = 0.488	0.999
Ischemic heart disease	25 (16.7)	10 (20.40)	15 (14.90)	χ^2^ = 0.733	0.391
Cancer	15 (10.0)	4 (8.20)	11 (10.90)	χ^2^ = 0.273	0.601
**Final outcomes**					
Face ulcer	17 (11.3)				
Tracheostomy	5 (3.3)	0 (0.00)	5 (5.00)	χ^2^ = 2.509	0.170
Discharged	50 (33.3)	47 (95.90)	3 (3.00)	χ^2^ = 128.26	< 0.001
Expired	100 (66.7)	2 (4.10)	98 (97.00)		

Abbreviations: BMI, body mass index; CRP, C-reactive protein; COPD, chronic obstructive pulmonary disease; ESR, erythrocyte sedimentation rate; GCS, Glasgow coma scale; LDH, lactate dehydrogenase; PCO_2_, partial pressure of carbon dioxide; RASS, Richmond Agitation-Sedation Scale; SpO_2_, oxygen saturation; VAS, visual analog scale; SF ratio, SpO_2_/ FIO_2_ ratio; ICU, intensive care unit.

^a^ Values are presented as mean ± SD or No (%) unless otherwise indicated.

The overall trend of the ROX index was downward, and the HACOR scale showed a downward trend within 6 hours of NIV initiation. However, between 6 hours and 12 hours after starting NIV, the trend became upward. Although SpO_2_ increased over time (P < 0.001), PCO_2_ decreased over the same period (P < 0.001). In terms of inflammatory parameters (lactate dehydrogenase [LDH], C-reactive protein [CRP], and erythrocyte sedimentation rate [ESR]), the mean of these parameters was higher than the normal threshold, indicating that most patients experienced some degree of inflammation.

The NIV failure rate was 67.3%, the mortality rate was 66.7%, and 3.3% of patients required a tracheostomy after NIV failure. According to nursing reports, 11.3% of patients developed face ulcers as a result of NIV masks.

Non-invasive ventilation failure had a significant relationship with patient mortality. Patients with NIV failure were more likely to expire (P < 0.001). This finding means that 97% of patients with NIV failure and 4.1% of non-NIV failure patients died. However, patients’ comorbidities (e.g., diabetes mellitus [DM] and hypertension [HTN]) did not show an association with NIV failure. The mean age of patients who failed NIV trials was significantly higher than non-failed patients (57 vs. 52) (P = 0.015). However, BMI was not associated with an increased risk of NIV failure. Non-invasive ventilation failure patients had a higher APACHE II score at the time of ICU admission (P < 0.001).

In terms of vital signs, pulse, and respiratory rate, there were statistically significant differences between the two groups of patients. Non-invasive ventilation failure patients had a higher pulse (97 vs. 87, P = 0.011) and respiratory rate (30 vs. 24, P < 0.001). Systolic blood pressure was not statistically different between the two groups of patients (P = 0.20). On average, both groups of patients had a GCS score of more than 14; however, as the GCS increment is only 1 scale point, it is challenging to identify significant differences between NIV failure and non-NIV failure patients.

In terms of inflammatory factors, only LDH was higher in patients with NIV failure (P < 0.001). Erythrocyte sedimentation rate and CRP did not show any significant differences between the two groups of patients. The SF ratio was slightly higher in non-NIV failure patients than in NIV failure patients (110 vs. 106, P = 0.013). The length of ICU stay did not differ statistically between the two groups of patients (P = 0.48).

**Table 2.** Univariate and Multivariate Logistic Regression Results ^a^

Univariate	β	OR (95% CI)	P-Value
**HACOR baseline**	- 0.163	0.850 (0.497 - 1.451)	0.551
**HACOR after 6 hours**	- 0.673	0.510 (0.170 - 1.533)	0.231
**HACOR after 12 hours**	2.266	9.641 (3.791 - 24.520)	< 0.0001
**ROX baseline**	2.611	13.607 (2.056 - 90.069)	0.007
**ROX after 6 hours**	- 0.265	0.768 (0.049 - 12.125)	0.851
**ROX after 12 hours**	- 5.367	0.005 (0.0001 - 0.077)	< 0.0001
**PCO2 baseline**	0.031	1.031 (0.979 - 1.086)	0.249
**PCO2 after 6 hours**	- 0.083	0.921 (0.847 - 1.000)	0.051
**PCO2 after 12 hours**	0.280	1.323 (1.191 - 1.469)	0.000
**LDH**	0.002	1.002 (1.001 - 1.003)	0.003
**Age **	0.032	1.032 (1.005 - 1.060)	0.018
**Multivariate for NIV failure ^a^**			
Age	0.025	1.025 (0.962 - 1.094)	0.445
HACOR baseline	- 0.780	0.458 (0.183 - 1.146)	0.095
HACOR after 6 hours	- 1.474	0.229 (0.051 - 1.027)	0.054
HACOR after 12 hours	2.910	18.363 (3.853 - 87.515)	0.000
PCO2 baseline	- 0.025	0.976 (0.874 - 1.090)	0.662
PCO2 after 6 hours	- 0.067	0.935 (0.785 - 1.113)	0.450
PCO2 after 12 hours	0.400	1.491 (1.127 - 1.973)	0.005
LDH	- 0.001	0.999 (0.996 - 1.002)	0.542
**Multivariate for in-hospital mortality**			
HACOR after 12 hours	0.753	2.123 (1.145 - 3.935)	0.017
PCO2 after 12 hours	0.273	1.314 (1.095 - 1.576)	0.003
ROX after 12 hours	- 1.507	0.222 (0.063 - 0.777)	0.019
Age	0.065	1.067 (1.000 - 1.138)	0.050
LDH	- 0.001	0.999 (0.997 - 1.002)	0.623
ESR	- 0.043	0.958 (0.921 - 0.996)	0.031
SF ratio	0.124	1.132 (1.029 - 1.247)	0.011

Abbreviations: OR, odds ratio; CI, confidence interval; ROX, ratio of oxygen saturation; ESR, erythrocyte sedimentation rate; LDH, lactate dehydrogenase; NIV, non-invasive ventilation; PCO_2_, partial pressure of carbon dioxide; SF ratio, SpO_2_/ FIO_2_ ratio.

^a^ It consists of all univariate variables but uses the ROX index for predicting NIV failure.

**Figure 2.**
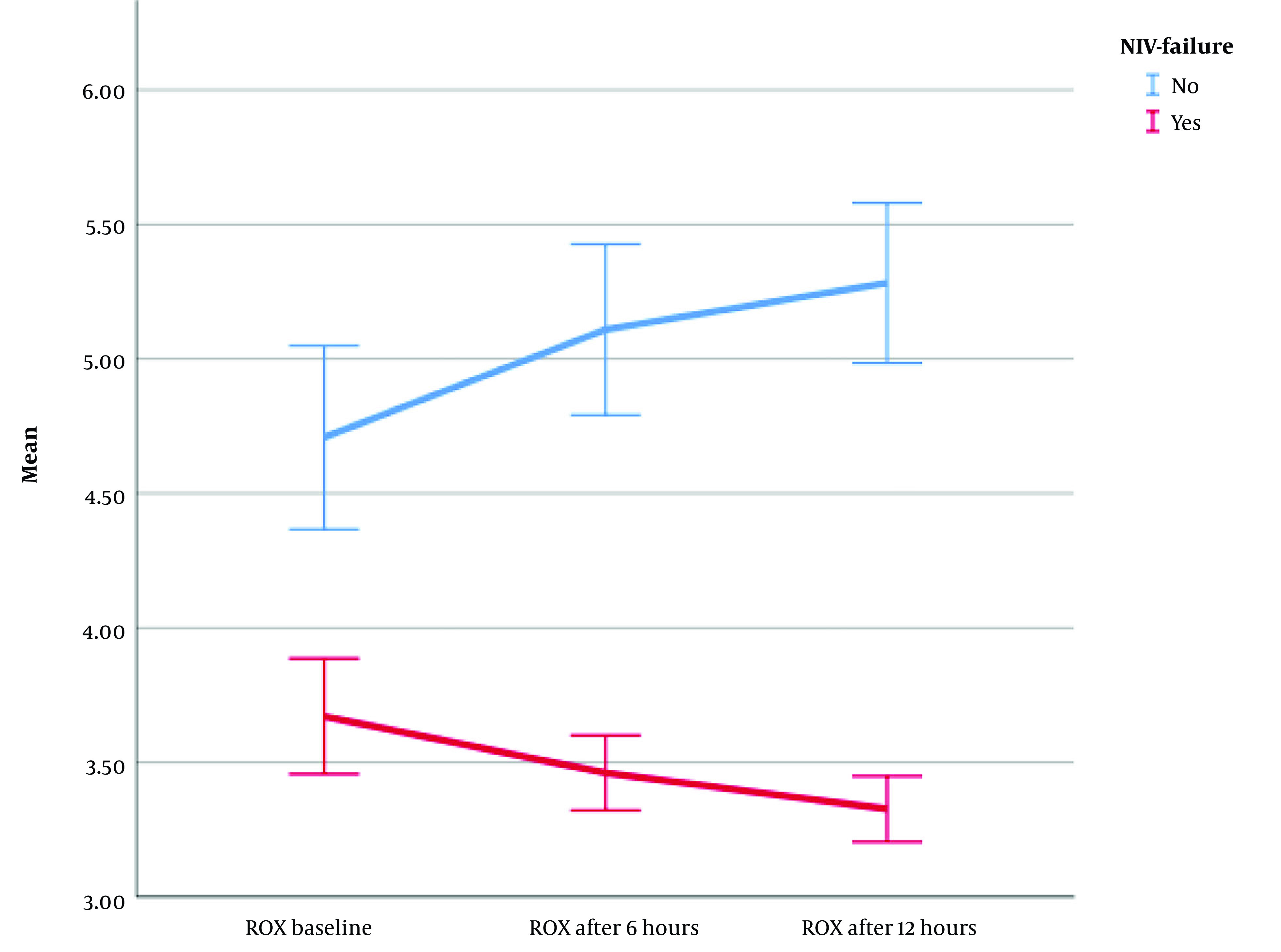
Comparison of ratio of oxygen saturation (ROX) index between two groups of patients over the time; (Abbreviations: ROX, ratio of oxygen saturation; NIV, non-invasive ventilation)

A repeated measures analysis of variance (ANOVA) (with Greenhouse-Geisser correction) determined that the mean ROX index differed statistically significantly between time points (F [1.216, 180.021] = 42.158, η2 = 0.22, λ = 0.75, P < 0.0001) (Figure 2). The ROX index decreased significantly over time in patients with NIV failure; however, it increased steadily in patients without NIV failure. Moreover, after NIV initiation, the ROX index increased in patients who successfully completed NIV but decreased in patients who developed NIV failure. Therefore, if the ROX index continuously decreases during the 6 to 12 hours after starting NIV treatment, it can be a predictor of failure.

**Figure 3.**
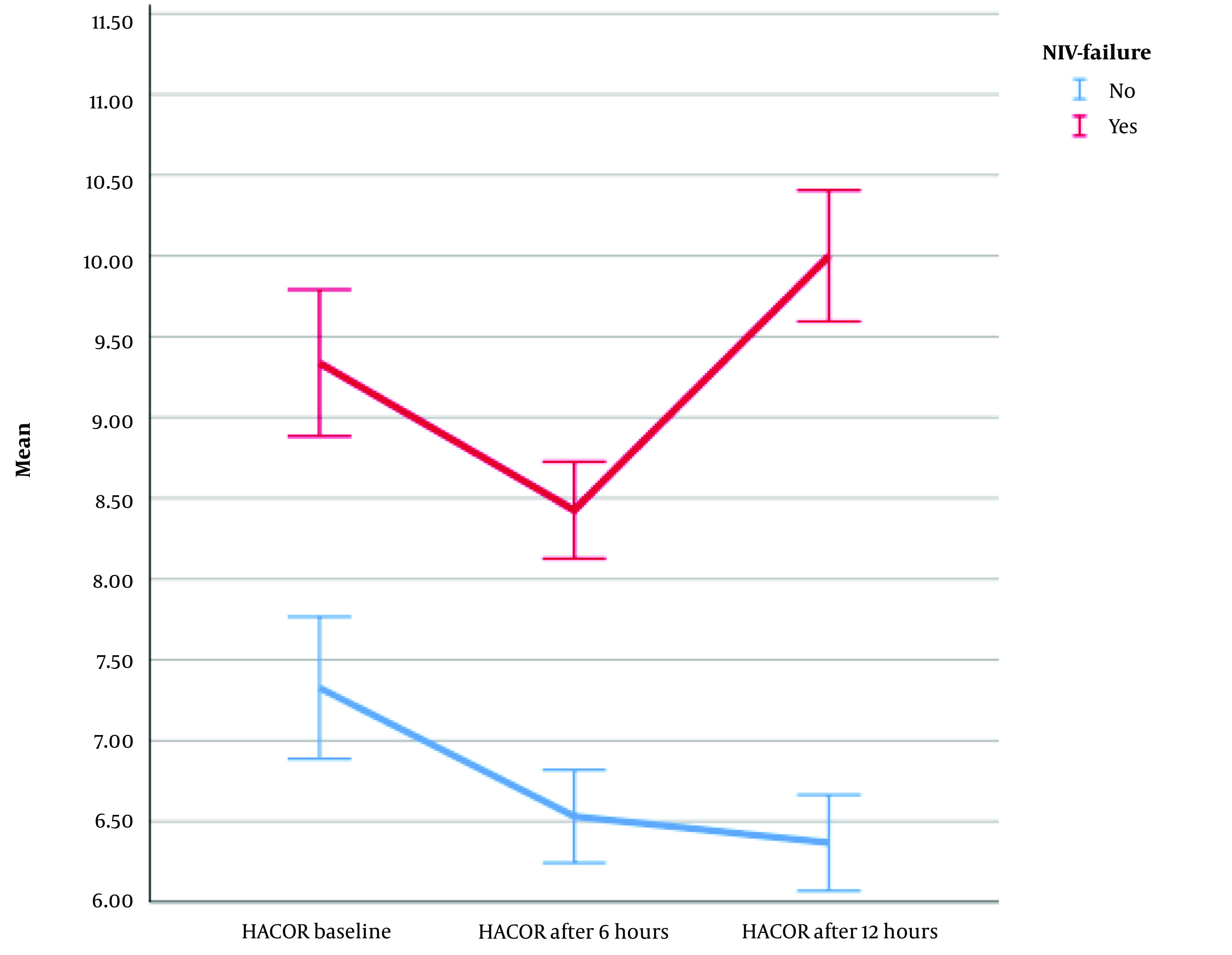
Comparison of HACOR between two groups of patients over the time; (Abbreviation: NIV, non-invasive ventilation)

Furthermore, the repeated measures ANOVA results (with Greenhouse-Geisser correction) revealed that the mean HACOR score differed statistically significantly between time points (F [1.910, 282.627] = 30.803, η^2^ = 0.17, λ = 0.65, P < 0.0001) (Figure 3). After the start of NIV in all patients, the HACOR score gradually decreased until 6 hours later. However, after 6 hours, the HACOR score of patients with ventilatory failure increased significantly; nevertheless, it gradually decreased in patients without ventilatory failure. Therefore, a continuous decrease in the HACOR score favors the success of the treatment, and an increase in the HACOR score after 6 hours can indicate treatment failure (NIV failure).

**Figure 4.**
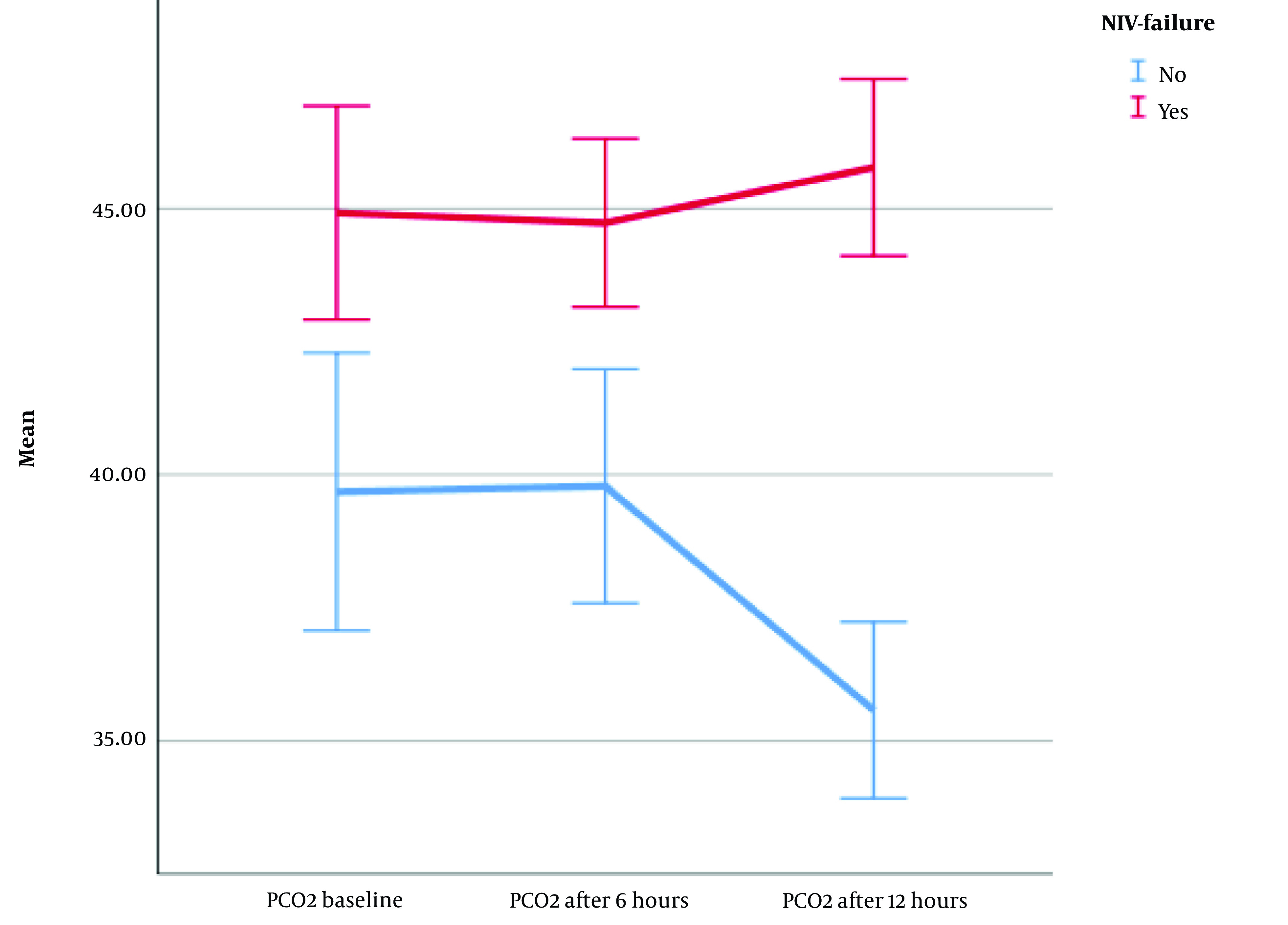
Comparison of partial pressure of carbon dioxide (PCO2) between two groups of patients over time; (Abbreviations: PCO_2_, partial pressure of carbon dioxide; NIV, non-invasive ventilation)

Finally, the mean PCO_2_ differed statistically significantly between time points (F [1.66, 245.693] = 7.962, η^2^ = 0.04, λ = 0.86, P = 0.001) (Figure 4). Although there was no significant difference in terms of PCO_2_ in the two groups of patients from the time of NIV initiation to 6 hours later, from 6 hours to 12 hours after the start of NIV, PCO_2_ was significantly different in patients without NIV failure. In patients without NIV failure, PCO_2_ steadily decreased until 12 hours after initiation. However, in NIV failure patients, from 6 to 12 hours later, this trend was slightly upward. Although there was no significant difference in the PCO_2_ of the patients from the start of treatment to 6 hours later, a decrease in PCO_2_ levels after 6 hours is indicative of treatment success; however, an increase or stability can predict failure.

The regression results (Table 2) revealed that in multivariate analysis, the HACOR scale and PCO_2_ levels 12 hours after NIV start are significant predictors of NIV failure. An increased HACOR score 6-12 hours after a NIV trial was associated with an 18-fold increase in the likelihood of NIV failure (OR = 18.363, P < 0.001). The partial pressure of carbon dioxide increased the likelihood of NIV failure by 1.491 times (OR = 1.491, P = 0.005) 6-12 hours after NIV initiation.

Finally, ESR (as an inflammatory index), SF ratio at ICU admission, HACOR, ROX, and PCO_2_ after 12 hours were significant predictors of mortality in patients receiving NIV. Increased HACOR (OR = 2.123, P = 0.017), PCO_2_ (OR = 1.314, P = 0.003), and decreased ROX index (OR = 0.222, P = 0.019) after 12 hours were significant predictors of in-hospital death. 

## 5. Discussion

The present study aimed to determine the predictors and associated factors of NIV failure among COVID-19 patients admitted to the ICU. In the current study, the rate of NIV failure was reported as 67.3%. Previous studies have reported varying rates of NIV failure among different patient populations, such as 50.2% in COPD patients (23), 20.6% in various patients (24), 50% in hypoxic patients, 25% in hypercapnic patients (25), 46% in acute respiratory distress syndrome (ARDS) patients (26), 61.1% in ARDS patients and 35% in non-ARDS patients (27), 66% in CAP patients (28), 15.5% in children with ARF (29), and 19.8% among respiratory failure patients (30). These findings suggest that the rate of NIV failure varies widely, ranging from 15.5% to 67.3% in non-COVID-19 patients.

Recently, it was reported that the rate of NIV failure in COVID-19 patients is 66.7% (31), which aligns closely with the present study’s findings. Therefore, it appears that NIV failure is strongly associated with primary respiratory diseases (CAP, COVID-19, and ARF), and COVID-19 patients in a critical care setting are more likely to experience NIV failure. In this study, all NIV-failed patients were intubated after a trial of NIV, indicating that NIV failure was defined as the need for intubation in COVID-19 patients in the ICU, consistent with previous research (5, 32).

The present study also aimed to conceptually differentiate between associated factors and predictors. It was demonstrated that standard scores are defined as predictors; nevertheless, demographic factors are considered associated factors. This can be explained by the fact that associated factors, such as gender, comorbidities, and age, are major, irreversible, and non-modifiable factors. This study showed that age was the only demographic factor associated with NIV failure, with patients older than 57 years being more likely to develop NIV failure. This finding is consistent with previous studies’ findings that have confirmed higher age as a risk factor for NIV failure and subsequent mortality (23, 24, 31, 33). However, BMI did not appear to be associated with NIV failure. It is worth noting that the population of the current study in terms of BMI might not be large enough to draw a strong conclusion. Previous research has linked obesity, especially comorbid obesity with a BMI greater than 35, to NIV failure and poor ICU outcomes (34, 35).

Comorbidities of the patients were not observed to be associated with NIV failure, which is consistent with the present study’s findings. A study (36) reported that the age-adjusted Charlson Comorbidity Index was not related to NIV failure. This finding suggests that NIV failure is not significantly associated with comorbidities, which aligns with the present study’s results. This lack of association might be attributed to the fact that some patients with comorbidities are hospitalized in emergency departments (EDs) or other wards and might pass away before being admitted to the ICU. In the current study, we specifically focused on patients admitted to the ICU. However, in a cohort study conducted in Michigan, USA, it was reported that higher age and a greater number of comorbidities were independent predictors of NIV failure in COVID-19 patients. The difference in the findings might be due to the fact that Imam et al. included all hospitalized COVID-19 patients, which differs from the present study’s population (33). Additionally, the aforementioned study had a larger sample size (N = 1 305). Nevertheless, further studies are needed to confirm these findings .

Predictors are defined as physiological parameters that can change based on the body’s physiology, such as the ROX index, which is calculated using SpO_2_ and respiratory rate. A recently published study (37) demonstrated that the HACOR scale can be a highly effective tool for predicting NIV failure in non-COPD patients receiving NIV. It was also reported (38) that the HACOR scale can be useful for predicting NIV failure in hypoxic patients with respiratory failure. One advantage of the HACOR scale is its simplicity in calculation at patients’ bedsides, and its reliability has been previously validated (20). It is worth noting that most of the studies mentioned above focused on non-COVID-19 patients in various hospital wards, including ICUs, which should be taken into consideration. All of these studies’ findings are consistent with the current study’s findings, suggesting that in terms of respiratory failure pathophysiology, NIV failure in COVID-19 patients might share similarities with ARF, COPD, and hypoxic patients.

According to a recent study (39), the HACOR scale is a reliable tool for predicting NIV failure in COVID-19 patients. Additionally, the present study demonstrated that the ROX index, when decreasing, can be another predictor of NIV failure in COVID-19 patients. According to a study (40), both the HACOR and ROX index are effective tools for predicting COVID-19 NIV failure, with similar accuracy and predictive value. Another study (41) suggested that the ROX index within 24 hours can be a useful predictor of high-flow nasal cannula (HFNC) and NIV success or failure.

The results of a meta-analysis indicated that the ROX index can serve as a valuable tool for predicting NIV failure among COVID-19 patients admitted to the ICU (42). The efficacy and reliability of the ROX index in predicting NIV failure in non-COVID-19 patients have also been demonstrated in previous studies (38, 43). Therefore, the ROX index appears to be a useful predictive tool for NIV failure in both COVID-19 and non-COVID-19 patients.

The findings of the current study revealed that, following the initiation of NIV in all patients, the HACOR score gradually decreased until 6 hours later. After 6 hours, the HACOR score significantly increased in patients with ventilatory failure; nonetheless, it continued to decrease in patients without ventilatory failure. However, the ROX index exhibited significant differences from the baseline in the two groups of patients. Specifically, patients who ultimately required intubation (NIV failure) showed a steady decline in their ROX index after the start of the NIV trial, although non-failure patients demonstrated an improvement in their ROX index following the initiation of NIV. A consistent decrease in the ROX index after the commencement of the NIV trial can be considered a predictor of NIV failure. Therefore, patients should be closely monitored, and both pharmacological interventions (e.g., bronchodilators or sedatives) and non-pharmacological interventions (e.g., prone positioning or chest physiotherapy) might be warranted.

Interestingly, PCO_2_ did not exhibit significant changes during the first 6 hours of the NIV trials. However, after 6 to 12 hours, patients with ventilatory failure showed a slight increase in PCO_2_; nevertheless, non-NIV failure patients experienced a significant decrease in PCO_2_. In comparison to the HACOR and ROX index, PCO_2_ appears to be a delayed predictor of NIV failure. Therefore, it might be considered after assessing the HACOR score and ROX index.

The current study demonstrated that the use of NIV in COVID-19 patients can effectively improve oxygen saturation in all patients, rendering it a beneficial intervention for severe respiratory failure in COVID-19 patients. However, it is crucial to note that this treatment should be employed for a trial period, typically ranging from 1 to 24 hours, based on physician judgment. This approach is taken because there is no international consensus regarding the utilization of NIV in COVID-19 patients (44, 45). Despite the widespread use of NIV in COVID-19 patients, this treatment does not appear to be associated with reduced complications and mortality (46). Therefore, further research is warranted to establish the efficacy of NIV and the optimal trial duration for COVID-19 patients.

Nursing care during an NIV trial (47) primarily involves eliminating environmental obstacles and striving to optimize ventilation and oxygenation in NIV patients (15). Although the prescription of NIV falls under the purview of intensivists (physicians), nurses play a crucial role in monitoring patients’ health and responding to NIV (17). One of the critical nursing responsibilities in caring for patients undergoing NIV is continuous monitoring during oxygen therapy. Nurses are responsible for monitoring the patient’s respiratory rate, level of consciousness, chest wall movement, use of accessory muscles, and comfort at 15-minute intervals following the initiation of NIV. This frequency can be reduced if the patient’s condition improves. Additionally, pulse oximetry and electrocardiography (ECG) monitoring should be maintained continuously during the initial 12 hours of NIV (48). Moreover, a lack of information (16) or insufficient knowledge about NIV can lead to inadequate attention to patients receiving this treatment. Therefore, it is of utmost importance for nurses to possess a comprehensive understanding of the NIV mechanism, nursing care during administration, and monitoring protocols. Nurses should be vigilant for changes in NIV failure predictors and promptly notify physicians when necessary.

### 5.1. Limitations

This study has two primary limitations. Firstly, it was a single-center study, and secondly, data collection was restricted to patient records. Additionally, the majority of the studied patients were critically ill, and further research involving patients with a mild to moderate degree of severity is required to validate the obtained findings.

### 5.2. Conclusions

In conclusion, the present study revealed a relatively high NIV failure rate (67.3%), emphasizing the need for preventive protocols. Advanced age (over 57 years) was associated with NIV failure; nevertheless, comorbidities, BMI, and gender did not show significant associations. The primary predictors of NIV failure included an increasing HACOR score after 12 hours, increasing PCO_2_ after 6 hours, and a decreasing ROX index following NIV initiation. These findings provide valuable insights for ICU practitioners and nurses regarding monitoring patients for NIV failure.

### 5.3. Implication for Practice

The present study’s findings underscore the significance of monitoring these predictors at patients’ bedsides. Healthcare professionals are recommended to consistently calculate and track the HACOR scale and ROX index, paying special attention to changes, such as an increase in the HACOR scale or a decrease in the ROX index. Furthermore, conducting further research with a larger sample size has the potential to offer more robust insights into NIV outcomes.

## Data Availability

The data used to support the findings of this study are available from the corresponding author upon request.
